# Genetic Diversity of *Pyrenophora tritici-repentis* Based on Virulence Phenotyping, Effector Gene Profiling, and SSR Markers

**DOI:** 10.3390/jof12100787

**Published:** 2026-10-20

**Authors:** Nina Mironenko, Nadezhda Kovalenko, Olga Baranova, Elena Gultyaeva

**Affiliations:** All Russian Institute of Plant Protection, Shosse Podbelskogo 3, Pushkin, St. Petersburg 1986608, Russiabaranova_oa@mail.ru (O.B.); eigultyaeva@gmail.com (E.G.)

**Keywords:** atypical isolates, population structure, races, Russia, tan (yellow) spot, *Triticum aestivum*, *Triticum durum*, Volga region, wheat

## Abstract

Tan spot is an economically important disease that occurs wherever wheat is grown in Russia. The variability of *Pyrenophora tritici-repentis* isolates collected in 2025 from common and durum wheat in the Volga region of the Russian Federation—namely Tatarstan, Penza, and Saratov—was characterised for virulence phenotypes, effector genes *ToxA*, *ToxB*, and *ToxC*, and SSR loci. A total of 74 isolates were studied. The *ToxA* gene was identified in 83.8% of the isolates, while *ToxB* was not detected; conversely, the *ToxC1* allele, one of the genetic determinants of PtrToxC, was present in all isolates. A discordance between phenotypic and molecular assays was noted for PtrToxA detection. Genetic diversity was examined using ten microsatellite (SSR) markers. After clone correction, 55 multilocus haplotypes were resolved. Isolates from common wheat in Tatarstan and Penza showed high similarity, in contrast to the Saratov isolates from durum wheat, which were significantly differentiated. The observed differentiation may be associated with host specialisation to durum wheat, but this hypothesis requires further validation with multi-year and genomic data.

## 1. Introduction

Bread wheat (*Triticum aestivum*) is one of the world’s most important cereal crops. However, its productivity is severely constrained by a variety of biotic factors, including fungal diseases, insect pests, and weeds. Tan spot (also known as yellow spot), incited by the necrotrophic fungus *Pyrenophora tritici-repentis*, ranks among the most prevalent and destructive wheat diseases globally, including Russian Volga regions. The Volga region, which encompasses 14 constituent entities of the Russian Federation—including the Republic of Tatarstan and the Penza and Saratov regions—is one of the main grain-producing regions. Wheat is the predominant crop in this region, accounting for 40% to 55% of the total sown area on average. Tan spot typically causes yield losses of 5–10% in wheat; however, under favourable conditions and on susceptible cultivars, losses can exceed 65% [1]. In the Saratov region, widespread disease occurrence has been documented since 2000 [2,3], and by 2019 disease severity had reached 80% on susceptible varieties [4].

Tan spot of wheat is caused by the homothallic ascomycete fungus *P. tritici-repentis* (*Ptr*), which produces specific exotoxins known as necrotrophic effectors (NEs). These include two protein toxins, PtrToxA and PtrToxB—controlled by the effector genes *ToxA* and *ToxB*—which induce necrosis and chlorosis on the leaves of susceptible varieties, respectively, and a secondary metabolite, PtrToxC (controlled by the *ToxC* gene), which also induces chlorosis. Unlike PtrToxA and PtrToxB, PtrToxC was characterised as a low-molecular-weight molecule more than 20 years ago [5]. However, the genetic control of PtrToxC remains unknown. One gene, designated *ToxC1*, has been cloned; it encodes a conserved hypothetical protein that is necessary but not sufficient for PtrToxC production [6]. Host plant sensitivity to these three NEs is governed by the *Tsn1*, Tsc2, and *Tsc1* genes, respectively [7]. PCR detection of *ToxC1* confirms only the presence of the gene and cannot demonstrate PtrToxC production, because *ToxC1* is necessary but not sufficient for toxin synthesis.

*Ptr* isolates are classified into distinct races according to their ability to elicit necrosis or chlorosis on the three standard wheat differentials: the cultivar Glenlea and lines 6B365 and 6B662 [8,9,10]. Based on the production of individual NEs or their combinations, eight races have been described, including race 4, which is nonpathogenic and does not produce any of the three known NEs. Interestingly, race 4 isolates harbour the *toxb* gene, a homologue of *ToxB* [11]. Moreover, in addition to the eight established races, mounting evidence points to the existence of further races with yet-unidentified NEs [7,12,13,14,15,16,17,18].

Although *Ptr* is homothallic, it displays substantial variation in virulence and molecular characteristics. Knowledge of the genetic diversity both within and among pathogen populations is fundamental for understanding population origins and migration dynamics, which is of critical importance for resistance breeding and the rational use of fungicides [19]. Surveillance of the racial structure of *Ptr* populations is necessary to evaluate the pathogen’s evolutionary capacity and to inform the development of wheat cultivars with long-lasting resistance. Host specialisation and spatial isolation are key factors driving the divergence of pathogen populations. These questions are critical for understanding the evolutionary dynamics of *Ptr* and for developing long-term strategies for tan spot management.

A range of molecular markers have been employed previously for genotyping *Ptr* isolates, including AFLP [20,21], RAPD [22], and SSR markers [23,24,25,26,27,28]. SSR markers have consistently been shown to be the most informative for population genetic studies of fungi, likely owing to their high mutation rates and rapid evolution within fungal genomes [29].

The aim of this study was to characterise the Volga populations of *P. tritici-repentis*, represented by three sample sets collected from common and durum wheat in 2025 from three distinct grain-producing regions within the Volga area of Russia, in terms of racial composition, the presence of the effector genes *ToxA, ToxB*, and *ToxC1*, and SSR marker diversity.

## 2. Materials and Methods

### 2.1. P. tritici-repentis Isolates Collections

The study comprised fungal isolates recovered from wheat leaves exhibiting tan spot symptoms during the 2025 growing season. These *Ptr* isolates were sampled from three regions of the Volga area in the Russian Federation—Tatarstan, Penza, and Saratov —as shown in Figure 1. A total of 25 isolates were recovered from leaves of *T. aestivum* varieties showing tan spot symptoms in Tatarstan, and 26 isolates were obtained from Penza. In Saratov, samples of *Ptr* were collected from durum wheat, from which 23 isolates were derived (Table S1). Based on the region of origin, the obtained tan spot isolates were collectively referred to as the Volga population.

Pure cultures of *Ptr* were recovered from symptomatic leaves following previously established protocols [30]. For all subsequent characterisation, genetically homogeneous monoconidial isolates were used. Species confirmation was carried out using both taxonomically relevant morphological features of conidia and PCR amplification with species-specific primers [31].

### 2.2. Race Identification

Race identification was performed by inoculating the first leaf of wheat seedlings with a conidial suspension from each isolate (3–5 × 10^3^ conidia/mL). Five plants of each differential line were used for inoculation. After inoculation, the plants were kept in darkness for 24 h at 100% humidity, after which they were placed under controlled conditions (22–23 °C, photoperiod 18 h light/6 h dark). On the 5th–6th day, the presence or absence of necrosis and chlorosis was recorded according to the scale of Lamari and Bernier [8]. If the reaction score was ambiguous, the experiment was repeated.

Race classification of the isolates was performed on the differential wheat genotypes Glenlea, 6B662, and 6B365, which carry the dominant susceptibility genes *Tsn1*, *Tsc2*, and *Tsc1*, respectively. These differentials respond to the corresponding necrotrophic effectors—PtrToxA, PtrToxB, and PtrToxC—encoded by the pathogen genes *ToxA*, *ToxB*, and *ToxC*, resulting in either necrosis or chlorosis. Race identification was carried out according to the standard protocol [8,32] (Table 1).

### 2.3. DNA Extraction and Amplification of Effector Genes

Monoconidial isolates were cultured on modified V8 medium [30]. DNA was extracted from seven- to ten-day-old mycelium using the CTAB (cetyl trimethyl ammonium bromide) protocol [33]. DNA quality and concentration were evaluated using a Nano-500 micro-spectrophotometer (AllSheng, Hangzhou, China), and the samples were subsequently diluted to a final concentration of 10 ng/µL in double-distilled deionized water.

Gene-specific primers were used to amplify the effector genes *ToxA*, *ToxB*, *ToxC1*, and *toxb*, as well as the chitin synthase gene *CHS-1*, which served as a positive control for the PCR reaction [6,14,31,34,35] (Table S2). PCR amplifications were carried out following the protocols recommended by the respective primer authors, with a total reaction volume of 25 μL. PCR products were resolved by electrophoresis on 1.7% agarose gels and visualised under UV light following ethidium bromide staining. Fragment sizes were determined using 100 or 50 bp DNA Ladder. Isolates of *P. tritici-repentis* that had been previously characterised as harbouring the *ToxB* and *toxb* genes [36,37] served as positive controls for the respective amplifications.

### 2.4. SSR Genotyping

Microsatellite (SSR) primers developed for the Pt-1C-BFP isolate (race 1) were employed for genotyping [24]. In the original study, SSR amplification products were resolved on both agarose and polyacrylamide gels. The same electrophoretic approach was used in the present work.

A set of ten SSR loci was used to genotype the 74 fungal isolates (Table S3). All PCR reactions were carried out in a C1000™ Thermal Cycler (Bio-Rad, Hercules, CA, USA). Each 25 µL reaction mixture contained 1× PCR buffer (without MgCl_2_), 2.5 mM MgCl_2_, 250 µM of each dNTP, 200 nM of each primer, 1.25 U of Taq DNA polymerase, and 20 ng of genomic DNA. The PCR programme comprised an initial denaturation step at 95 °C for 5 min, followed by 35 cycles of 95 °C for 30 s, 55 °C for 20 s, and 72 °C for 30 s, with a final extension at 72 °C for 10 min. Electrophoretic separation of the amplification products was performed on 1.7% agarose gels and 6% polyacrylamide gels stained with ethidium bromide; gels were run at 100 V for 3 h in 0.5× TBE buffer. Amplicon sizes were estimated using the GeneRuler™ 50 bp DNA molecular weight ladder (Thermo Fisher Scientific, Waltham, MA, USA).

### 2.5. Data Analysis

For the analysis of population genetic structure based on SSR markers, a binary matrix was constructed from the sizes of the amplified fragments. Fragments of identical length were scored as the same allele for each SSR locus.

All statistical analyses were carried out using GenAlEx 6.5 version [38]. Intrapopulation genetic diversity at SSR loci was assessed using the following parameters: mean number of alleles per locus (Na), effective number of alleles (Ne), expected (He) and observed (Ho) heterozygosity, and Shannon’s diversity index (I). Genetic differentiation among regional *P. tritici-repentis* populations was estimated using PhiPT values derived from AMOVA (analysis of molecular variance) with 999 permutations. According to the widely used interpretation, PhiPT values in the range of 0.05–0.15 correspond to moderate genetic differentiation, whereas values of 0.15–0.25 indicate significant differentiation.

Genetic diversity at each polymorphic SSR locus was estimated using the formula Hi = 1 − ∑pi^2^, where pi denotes the frequency of the ith allele. The allele size range for each locus was calculated as the difference between the largest and smallest amplified fragments. Mean gene diversity was computed on a clone-corrected dataset (i.e., after removing clonal genotypes).

The clonal fraction (CF) of the population was derived according to the equation CF = 1 − (*nu*/*n*), where *nu* represents the number of distinct multilocus genotypes and *n* is the total sample size [39].

Multidimensional scaling of genetic distances was performed using principal coordinate analysis (PCoA) with Nei’s genetic distance matrix.

A neighbour-joining (NJ) dendrogram illustrating the genetic relationships among *P. tritici-repentis* isolates was generated using MEGA 11 (https://www.megasoftware.net/). The pairwise distance matrix for phylogenetic tree construction was calculated from a binary matrix without clone correction using the PhyloM software version 1.0 (https://www.mdpi.com/2075-1729/12/5/719) (accessed 10 August 2026).

## 3. Results

### 3.1. Racial Structure of the P. tritici-repentis Population

A total of 74 isolates were recovered from symptomatic leaf samples collected in the Volga region of Russia: 25 from Tatarstan, 26 from Penza, and 23 from Saratov. The samples from Tatarstan and Penza were obtained from common wheat (*T. aestivum*), whereas those from Saratov originated from durum wheat (*T. durum*). Species identity of all monoconidial isolates was confirmed by PCR using the species-specific primer pair PtrUniqueF2/PtrUniqueR2 [31].

Race classification of the 74 *P. tritici-repentis* isolates was performed based on their virulence reactions on the three differential genotypes: cv. Glenlea and lines 6B662 and 6B365. Of these, 27 isolates were assigned to race 1, 12 to race 2, and 10 to race 3. Fifteen isolates were avirulent on all three differentials and were classified as race 4. A single isolate, identified as race 6, was avirulent on Glenlea but elicited chlorosis on both 6B662 and 6B365. Three isolates induced necrosis on Glenlea and chlorosis on 6B662, but showed no reaction on 6B365, and were therefore designated as race 7. Six isolates, which caused necrosis on Glenlea and chlorosis on both 6B662 and 6B365, were classified as race 8 (Table 2). No race 5 isolates were detected in the collection.

### 3.2. Identification of NE Genes

A total of 74 isolates from the Volga population were screened for the presence of the effector genes *ToxA, ToxB, ToxC1*, and *toxb*. The *ToxA*-specific primers amplified the expected 573 bp fragment in 62 isolates. Among these, 39 ToxA^+^ isolates—comprising races 1, 2, 7, and 8—elicited necrosis on the differential cultivar Glenlea (Table S1), whereas the remaining 23 ToxA^+^ isolates (races 3, 4, and 6) did not produce a necrotic reaction on Glenlea. A representative duplex PCR amplification of the *ToxA* gene fragment together with the *CHS-1* housekeeping gene [34] is presented in Figure 2.

All isolates were screened for the presence of the *ToxA* open reading frame using specific primers [14]. The results obtained with these primers were fully consistent with those from the Ta51F/Ta52R primer pair [34] (Table S1). The *ToxB* gene was not amplified in any of the isolates tested, using two independent primer pairs reported by different research groups [14,34,35] (Table S1). By contrast, the *toxb* gene—a homologue of *ToxB* or a non-functional allele thereof—was detected in three isolates from the Saratov collection (Sar12-6, Sar14-1, and Sar15-3) using gene-specific primers developed by different authors [14,34,35] (Table S1). For the identification of the *ToxC1* gene, which encodes a conserved hypothetical protein required but not sufficient for PtrToxC production [6], primers M4_13157F and M4_13157R were employed (Table S2). The diagnostic 505 bp *ToxC1* amplicon was present in all 74 fungal isolates examined.

### 3.3. Genetic Diversity of the Volga P. tritici-repentis Collections Based on SSR Markers

To assess the genetic variability of *Ptr* collections, ten polymorphic microsatellite (SSR) loci distributed on different chromosomes were selected. Genotyping of the 74 isolates was carried out using primers specific to each SSR locus [24] (Table S3).

The number of alleles per locus varied from 2 to 11 (Table 3), with a mean allelic richness of 5.40 ± 0.94 alleles per locus. The amplified fragment sizes ranged between 150 bp and 550 bp (Table S4). Gene diversity, estimated from the clone-corrected dataset, was consistently high across all three population samples. The mean gene diversity values were H = 0.60 and 0.63 for the Tatarstan and Penza samples, respectively, while the Saratov sample exhibited a slightly lower value of H = 0.49 (Table 3). Allele frequencies showed marked differences among the three population samples, and certain alleles were unique to individual samples. For example, alleles of 300 bp and 350 bp at locus PtrSSR-07, and alleles of 350 bp and 400 bp at locus PtrSSR-15, were detected exclusively in the Saratov population. The Tatarstan population possessed private alleles at loci PtrSSR-07 (190 and 200 bp) and PtrSSR-15 (200 bp), while the Penza population was distinguished by alleles of 350, 500, and 550 bp at locus PtrSSR-16 (Table S4). The range of allelic size variation was determined for each locus across the three population samples (Table 3). The mean allele sizes per locus were statistically comparable among the samples: 80.00 ± 20.17 bp (Tatarstan), 101.00 ± 26.93 bp (Penza), and 80.00 ± 20.76 bp (Saratov).

Each isolate was assigned a multilocus haplotype (MLHT) consisting of ten alleles, one from each SSR locus examined. The clonal fraction (Cf) was estimated for each population sample: 0.16 for Tatarstan, 0.31 for Penza, and 0.26 for Saratov, resulting in an overall Cf of 0.23 for the Volga population. Following clone correction, all population samples consisted exclusively of unique haplotypes. Analysis of molecular variance (AMOVA) revealed that 83% of the SSR variation resided within populations, whereas 17% was attributable to differentiation among population samples. From the clone-corrected data matrix, a total of 55 multilocus haplotypes (MLHT) were identified across the three samples. Of these, 43 genotypes were represented by single isolates, while the remaining 31 isolates belonged to 12 clonal lineages (designated A–L), each comprising 2 to 5 isolates sharing identical genotypes. Importantly, each clonal lineage was recovered from a single sampling site and from the same host cultivar (Table S1). The genetic diversity parameters of the Tatarstan and Penza populations were comparable, and both were higher than those observed for the Saratov population (Table 4).

### 3.4. Molecular Diversity and Genetic Differentiation Among Pyrenophora tritici-repentis Populations from the Volga Region

Genetic differentiation among the three population samples was assessed by AMOVA using a binary data matrix, with PhiPT (Fst) indices calculated for pairwise comparisons. The Tatarstan and Penza populations exhibited the highest degree of molecular similarity (Fst = 0.078, *p* ≤ 0.001). In contrast, the Saratov population was significantly differentiated from both Tatarstan and Penza, with Fst values of 0.178 and 0.144, respectively (*p* ≤ 0.001). When the combined sample of isolates from common wheat (Tatarstan and Penza) was compared with the Saratov population, which originated from durum wheat (Table S1), a moderate level of genetic differentiation was observed (Fst = 0.135, *p* ≤ 0.001).

AMOVA revealed that 83% of the SSR variation was distributed within populations, whereas 17% was attributable to differentiation among population samples. The genetic relationships among the *Ptr* isolates were explored using NG clustering and principal coordinate analysis (PCoA) (Figure 3 and Figure 4). Figure 3 shows the dendrogram of genetic relationships for the entire isolate collection, including clonal genotypes, while Figure 4 displays the relationships based solely on isolates with unique haplotypes (55 in total).

### 3.5. Racial Differentiation Among Pyrenophora tritici-repentis Populations from the Volga Region

The phenotypic reactions of isolates on the three wheat differential genotypes can be expressed as a binary matrix, where ‘1’ denotes the presence and ‘0’ the absence of necrosis or chlorosis on each differential. The three binary traits corresponding to the production of the necrotrophic effectors PtrToxA, PtrToxB, and PtrToxC generate eight possible combinations, which correspond to the eight known races of *P. tritici-repentis* (Table 1). With respect to racial diversity, the highest level of differentiation was observed between the Tatarstan and Penza populations (Fst = 0.124, *p* ≤ 0.01). In contrast, the Saratov population exhibited considerable similarity to both populations (Fst = 0.053, *p* ≤ 0.071 and Fst = 0.030, *p* ≤ 0.148).

A Mantel test was performed to compare the genetic distance matrix derived from SSR markers with the virulence phenotype matrix. The analysis revealed a significant negative correlation (r = 0.96; t (the test statistic) = −3.336 (df = 1); *p* ≈ 0.185). suggesting that isolates with greater genetic divergence at neutral SSR loci tend to exhibit less phenotypic variation in virulence.

## 4. Discussion

The Volga population exhibits considerable race diversity. The frequency of isolates belonging to the dominant race 1 was 36.5%, in contrast to the Australian population, where race 1 comprised 96% of isolates [28], and the Tunisian population, in which this race was entirely absent [14].

According to the race classification scheme (Table 1), strains designated as races 1, 2, 7, and 8 elicit necrosis on Glenlea leaves (nec^+^) and, in accordance with the inverse gene-for-gene model governing the *Triticum aestivum–Pyrenophora tritici-repentis* pathosystem [7], are expected to possess the *ToxA* gene. These isolates were assigned the phenotypic designation nec^+^ToxA^+^. In contrast, isolates of races 3, 4, 5, and 6 do not induce necrosis (nec^−^) and are predicted to lack *ToxA*; their phenotype was recorded as nec^−^ToxA^−^. Molecular screening for the *ToxA* gene (Table S1, Table 2) showed that among the 48 nec^+^ strains, 39 (81.2%) were ToxA-positive, consistent with their capacity to produce PtrToxA and induce necrosis on Glenlea. Notably, the remaining nine nec^+^ strains (18.8%) were *ToxA*-negative (phenotype nec^+^ToxA^−^), indicating that they likely induce necrosis via a toxin other than PtrToxA.

It has been widely reported that *Ptr* isolates inducing necrosis in the absence of the *ToxA* gene are inconsistent with the canonical race classification system and have consequently been designated as ‘atypical’. This inconsistency is hypothesised to result from the presence of additional, as yet uncharacterised, necrotrophic effectors (NEs) produced by the fungus [12,13,14,15,16,17,18,40]. As early as 2006, isolate OS3 from southern Oregon was reported to lack the *ToxA* gene while phenotypically behaving as race 8 [34]. Subsequently, individual atypical *Ptr* isolates were identified in Algeria [11], Argentina [41], Australia [42], and Russia [16]. Over the past decade, an increasing number of reports have indicated a rise in the proportion of atypical isolates within *Ptr* populations. Notably, in a North African population, the frequency of atypical isolates reached 44%.

Conversely, among the 26 nec^−^ strains of races 3, 4, and 6, 23 isolates (88.5%) were shown to harbour the *ToxA* gene, although it was not expressed under the conditions tested. These strains were also assigned to the ‘atypical’ category and designated as nec^−^ToxA^+^. Collectively, comparison of phytopathological and molecular assays for the detection of PtrToxA, PtrToxB, and PtrToxC and their corresponding genes in *Ptr* isolates revealed notable discrepancies. These findings highlight the limitations of the current race classification system and are in agreement with earlier reports.

The ‘atypical’ *Ptr* strains of the nec^+^ToxA^−^ phenotype identified in the present collection may involve additional or alternative necrotrophic effectors that cause necrosis on wheat leaves, mimicking the effect of PtrToxA. These strains pose a challenge for tan spot resistance breeding, as the genetic basis of the novel toxins and the corresponding wheat susceptibility genes remain largely unknown. Compared with other Russian populations previously examined by our group [18], the Volga population is characterised by a substantially higher frequency of isolates (over 30%) carrying the *ToxA* gene with impaired expression. In these isolates, the *ToxA* gene was detected by PCR but was not expressed under the conditions tested; this phenotype cannot be attributed to a specific mechanism based on PCR data alone. In contrast, in Russian populations surveyed during 2023–2025, the proportion of such isolates was only 16% [18]. The underlying causes of this phenomenon warrant further study. Elucidation of the underlying mechanism will require sequencing of the *ToxA* locus and ORF, promoter and indel analysis, and transcript- or protein-level evidence.

In the present study, we examined a collection of *Ptr* isolates recovered from infected leaves of two wheat species—*T. aestivum* and *T. durum*—cultivated in the Volga region of Russia. The isolate collection comprised three population samples: 51 isolates derived from common wheat (25 from Tatarstan and 26 from Penza) and 23 isolates obtained from durum wheat leaves grown in Saratov. All three locations (Tatarstan, Penza, and Saratov) share a temperate continental climate with well-defined seasonal variation. Nevertheless, owing to the considerable latitudinal gradient from north to south, the climate becomes increasingly arid and warmer towards the Saratov region.

Comparative analysis of molecular variation among the isolates in the present collection revealed that the Tatarstan and Penza samples, both recovered from common wheat, could be merged into a single population (Fst = 0.078, *p* < 0.001). By contrast, the Saratov sample, obtained from durum wheat, exhibited significant genetic differentiation from both Tatarstan (Fst = 0.178, *p* < 0.001) and Penza (Fst = 0.144, *p* < 0.001). Moreover, the Saratov isolates displayed lower genetic diversity than the Tatarstan and Penza samples for all population diversity indices examined. The divergence of the Saratov sample may be associated with climatic conditions and host-mediated selection, but our single-year data cannot distinguish between these factors. Multi-year sampling is needed to confirm whether this pattern is consistent. Indeed, when the Saratov isolates from durum wheat were compared with the combined common-wheat sample (Tatarstan + Penza), the genetic differentiation was substantial (Fst = 0.135, *p* ≤ 0.001). The clonal fractions observed within each population sample probably arose through selection of isolates on particular host cultivars. For each cultivar, isolates with identical haplotypes formed clonal lineages strictly associated with that cultivar, based on observations from the 2025 collection. The clonal fraction was notably higher in the Penza (Cf = 0.31) and Saratov (Cf = 0.26) samples, while it was substantially lower in the Tatarstan sample (Cf = 0.16). Notably, all 12 clonal lineages were restricted to a single sampling site and host cultivar (Table S1); however, in half of these clones, the constituent isolates exhibited different race designations.

The mean gene diversity estimates for the Volga population samples ranged from 0.49 to 0.63. These values are comparable to those reported for five populations from five continents (Asia, Europe, Australia, South America, and North America; Hs = 0.31–0.56) [24], as well as to an Australian population sampled from a single field in Western Australia in 2021 (H = 0.548) [28].

Analysis of race composition revealed differences among population samples in the frequencies of dominant races. In the Tatarstan *Ptr* sample, race 1 (PtrToxA, PtrToxC) comprised 56% of isolates. In Penza, race 2 (PtrToxA), races 3 (PtrToxC) and 4 (producing none of the known NEs) represented 23.1%, 30.8% and 23.1% of isolates, respectively, whereas in Saratov, race 1 (47,8%) and race 4 (26.1%) were the most frequent. The NG dendrogram showed clustering of isolates primarily by geographic origin and, more specifically, by host cultivar. This pattern was particularly evident for Saratov isolates recovered from two durum wheat cultivars, Saratovskaya Zolotistaya and Pamyati Vasilchuka, as well as for a portion of the Penza isolates. Principal coordinate analysis (PCoA) confirmed the close genetic relatedness between Tatarstan and Penza isolates, while revealing a distinct cluster comprising a subset of Saratov isolates. The negative correlation between SSR markers and virulence, as indicated by the Mantel test, is consistent with our observations of racial diversity within certain clones and supports the conclusion that no significant association was detected between neutral SSR markers and virulence traits. However, we present this result as a preliminary observation rather than a definitive relationship.

In the Volga population, no significant association was found between the geographic origin of the isolates and their race affiliation. This finding is in agreement with observations reported for *Ptr* populations from Canada, the Caucasus, the Fertile Crescent, and North Africa [23,26,43], as well as for Australian and European populations [28].

## 5. Conclusions

In the Volga population of *Ptr*, race 1 was dominant (36.5%), while races 2, 3, 4, and 8 occurred at frequencies of 16.2%, 13.5%, 20.3%, and 8.1%, respectively. Races 6 and 7 were each represented by a single isolate, and race 5 was not detected. Race identification of *Ptr* isolates was based on phenotypic reactions, and PCR of effector genes was used to supplement and refine the characterisation of the isolates.

In the Volga population, 83.8% of isolates harboured the *ToxA* gene. Among these, 10.8% were atypical isolates of the nec^+^ToxA^−^ phenotype, which may involve additional or alternative necrotrophic effectors, and 31.1% exhibited the nec^−^ToxA^+^ phenotype, characterised by the presence of a non-functional *ToxA* gene. The *ToxB* gene was not detected in any isolate, whereas the *ToxC1* gene was universally present. Analysis of molecular variance (AMOVA) revealed substantial genetic differentiation among the three population samples, which, given their geographic separation and distinct host genotypes, represent subpopulations with specific differences in both race composition and allelic diversity at neutral SSR markers. Because this study is based on a single growing season, all evolutionary inferences should be regarded as tentative. The results presented here provide a baseline for future monitoring, whereas conclusions regarding migration, origin, and host specialisation will require additional years of sampling and, ideally, genomic data. The ‘atypical’ Ptr strains of the nec^+^ToxA^−^ phenotype identified in the present collection likely produce novel exotoxin(s), possibly involving additional or alternative necrotrophic effectors that cause necrosis on wheat leaves and mimic the effect of PtrToxA. These strains pose a challenge for tan spot resistance breeding, as the genetic basis of the novel toxins and the corresponding wheat susceptibility genes remain largely unknown.

## Figures and Tables

**Figure 1:**
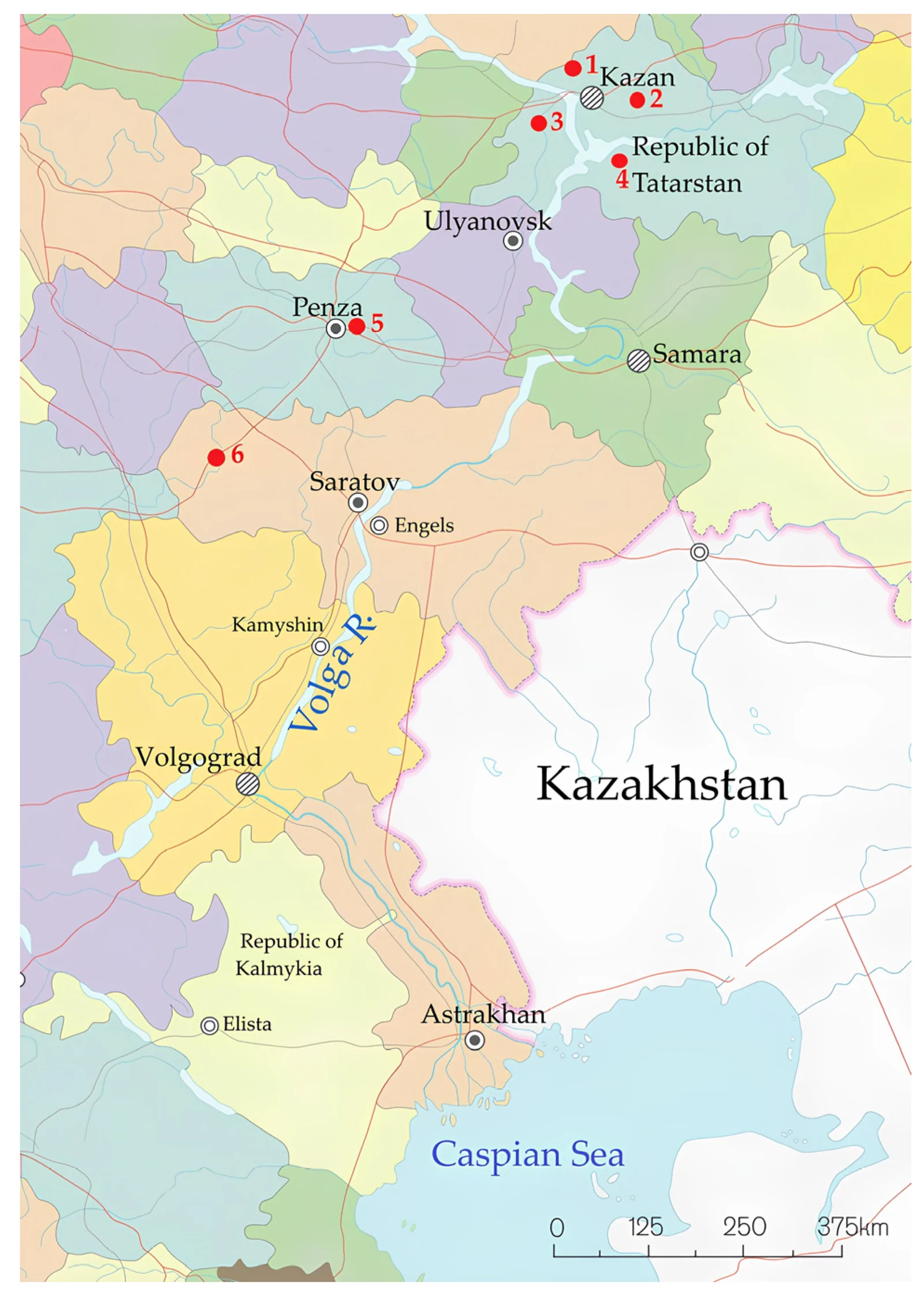
Geographic locations of *Pyrenophora tritici-repentis* sampling sites in the Volga regions of the Russian Federation: Tatarstan (sites 1–4: Vysokogorsky, Tyulyachinsky, Verkhneuslonsky, and Chistopol districts), Penza (site 5), and Saratov (site 6, Arkadak District).

**Figure 2:**
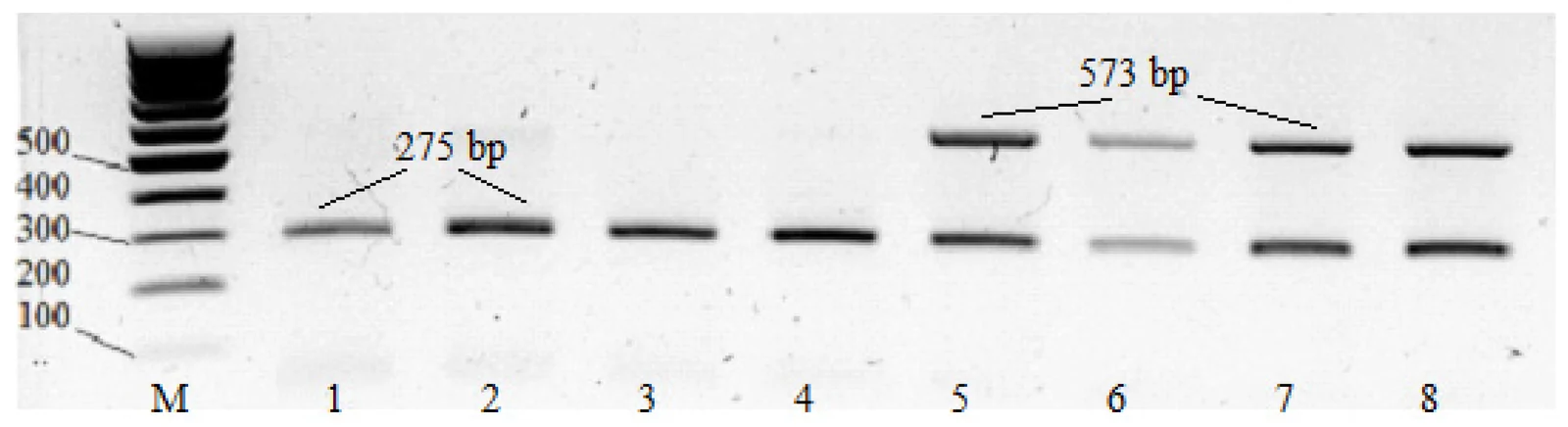
Duplex PCR amplification of the *CHS-1* housekeeping gene and the *ToxA* effector gene in *P. tritici-repentis* isolates from the Saratov population. Lane M—DNA ladder. Expected fragment sizes: *CHS-1*—275 bp, *ToxA*—573 bp. 1–8—isolates of *Ptr* from Saratov sample.

**Figure 3:**
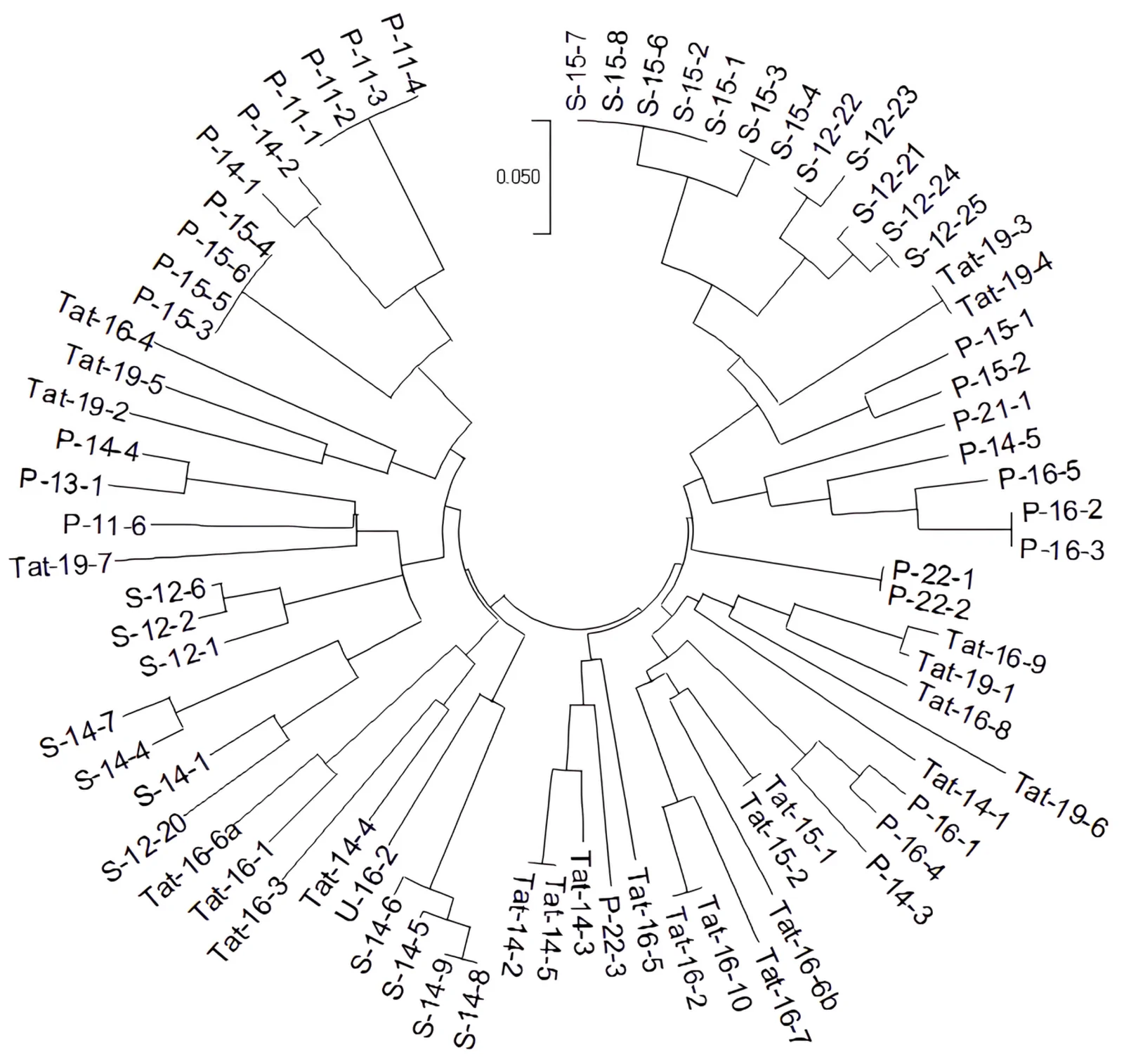
Genetic relatedness among *Pyrenophora tritici-repentis* isolates from three populations revealed by SSR markers: Tat (Tatarstan), P (Penza), and S (Saratov). Details on isolate provenance are given in Table S1.

**Figure 4:**
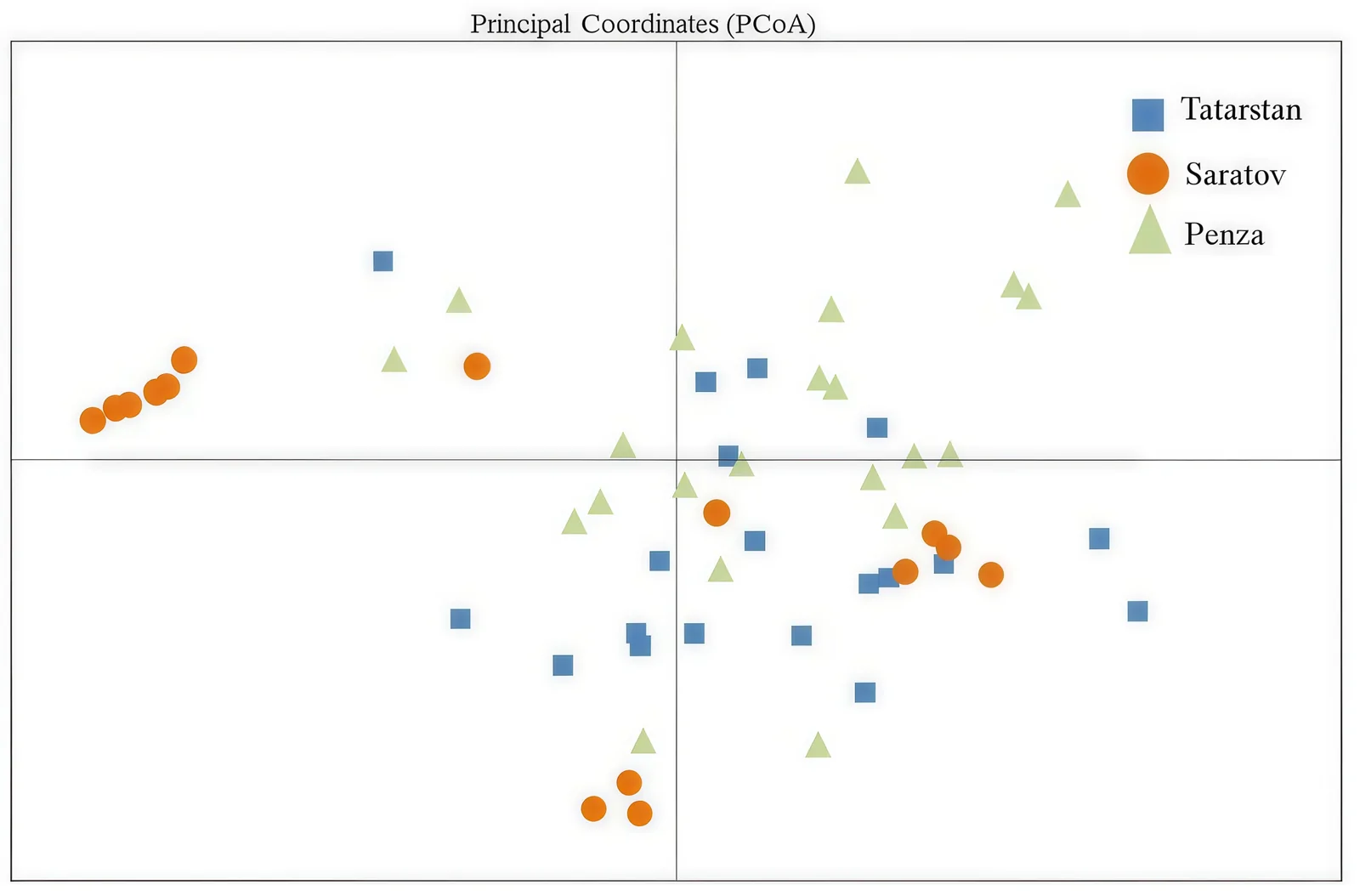
Principal coordinate analysis (PCoA) plot of *Pyrenophora tritici-repentis* isolates from three Volga populations, based on Nei’s genetic distance matrix and computed with GenAlEx.

**Table 1:** Characteristics of *P. tritici-repentis* races based on their ability to produce exotoxins (adapted from [32]).

Differential	Abbreviation of Races, Producing Exotoxins							
	1 AC *	2 A	3 C	4 -	5 B	6 BC	7 AB	8 ABC
Glenlea	nec	nec	-	-	-	-	nec	nec
6B662	-	-	-	-	cl	cl	cl	cl
6B365	cl	-	cl		-	cl	-	cl

* Abbreviations of races and exotoxin production profiles: AC—PtrToxA, PtrToxC; A—PtrToxA; C—PtrToxC; B—PtrToxB; BC—PtrToxB, PtrToxC; AB—PtrToxA, PtrToxB; ABC—PtrToxA, PtrToxB, PtrToxC. Symptoms: nec—necrosis on cultivar Glenlea (caused by PtrToxA); cl—chlorosis on line 6B662 (caused by PtrToxB) and on line 6B365 (caused by PtrToxC); ‘-’—no symptoms.

**Table 2:** Racial composition of the Volga population samples of *Pyrenophora tritici-repentis* in 2025 and the occurrence of ToxA^+^ strains.

Origin of *Ptr* Collections	Total Strains/ ToxA^+^	Number of Strains of Each Race/Number of ToxA^+^ Strains							
		1 AC *	2 A	3 C	4 No Exotoxins	5 B	6 BC	7 AB	8 ABC
Tatarstan	25/21	14/12	3/2	1/1	3/2	0	0	1/1	3/3
Penza	26/25	2/2	6/6	8/8	6/5	0	1/1	0	3/3
Saratov	23/16	11/6	3/3	1/1	6/5	0	0	2/1	0
Total isolates	74/62	27/20	12/11	10/10	15/12	0	1/1	3/2	6/6

* The toxins produced are designated as shown in Table 1.

**Table 3:** Gene diversity of *Pyrenophora tritici-repentis* population samples from the Volga region at ten microsatellite loci.

Locus	Size Range (bp)	Number of Alleles in Population Samples			Total Number of Alleles per Locus	Gene Diversity at Individual Loci (1) and Maximum Allele Size Difference (bp) of SSR Loci (2) in Population Samples					
						Tatarstan		Penza		Saratov	
		Tatarstan	Penza	Saratov		1	2	1	2	1	2
PtrSSR-01	239–250	2	2	2	2	0.49	20	0.48	20	0.49	20
PtrSSR-03	250–300	4	4	1	4	0.67	50	0.58	50	0.0	0
PtrSSR-05	200–280	5	6	4	6	0.71	50	0.77	80	0.68	20
PtrSSR-07	170–400	7	5	7	11	0.76	210	0.69	110	0.81	190
PtrSSR-09	250–300	3	3	3	3	0.48	50	0.54	50	0.57	50
PtrSSR-12	250–300	3	3	2	3	0.65	50	0.48	50	0.22	50
PtrSSR-13	300–450	3	5	5	6	0.28	20	0.70	150	0.75	150
PtrSSR-14	200–250	3	3	2	3	0.56	50	0.54	50	0.38	20
PtrSSR-15	150–400	3	5	4	8	0.65	100	0.73	150	0.56	150
PtrSSR-16	200–550	5	6	3	8	0.71	200	0.78	300	0.47	150
Mean		3.80	4.20	3.30	5.40	0.60	80.00	0.63	101.00	0.49	80.00
±SE		0.42	0.44	0.54	0.94	0.04	20.17	0.03	26.93	0.07	20.76

**Table 4:** Genetic diversity indices of *Pyrenophora tritici-repentis* collections from the Volga region estimated using SSR markers.

Origin of *Ptr* Collections	*N*	Na	Ne	I	He	uHe	Poly Morphic Loci, %
Tatarstan	25	1.455 ± 0.121	1.244 ± 0.036	0.268 ± 0.030	0.164 ± 0.012	0.167 ± 0.021	72.73
Penza	26	1.564 ± 0.112	1.228 ± 0.034	0.262 ± 0.029	0.157 ± 0.020	0.160 ± 0.020	78.18
Saratov	23	1.182 ± 0.133	1.208 ± 0.038	0.221 ± 0.031	0.136 ± 0.021	0.139 ± 0.022	58.18
Total Volga	74	1.400 ± 0.071	1.227 ± 0.021	0.250 ± 0.017	0.152 ± 0.012	0.155 ± 0.012	69.70 ± 5.97

*N*—number of isolates; Na—number of different alleles; Ne—effective number of alleles; I—Shannon’s Information Index; He—expected heterozygosity; uHe—unbiased expected heterozygosity.

## Data Availability

The data used in this study are available from the corresponding author upon reasonable request.

## Supplementary Materials

The following supporting information can be downloaded at https://www.mdpi.com/article/10.3390/jof12100787/s1. Table S1. Isolates of the Volga population of *P. tritici-repentis* (Ptr) included in the study; Table S2. Sequences of primers used in the work; Table S3. Primers for microsatellites of *Pyrenophora tritici-repentis* used in the work; Table S4. Observed allele frequencies of ten microsatellite loci in samples of the Volga population of *Pyrenophora tritici-repentis*.
