# Supplementary material for: Genetic Diversity of Pyrenophora tritici-repentis Based on Virulence Phenotyping, Effector Gene Profiling, and SSR Markers
Source: J Fungi (Basel). 2026 Oct 20;12(10):787. doi: 10.3390/jof12100787 (PMC13705791; doi:10.3390/jof12100787)
Supplement: Supplementary file 1 [file jof-12-00787-s001.zip › Table S1.pdf]

Table S1. *Isolates of the Volga population of P. tritici-repentis (Ptr) included in the study.*

Isolate	Origin	Host	Reaction of wheat genotypes to infection with *Ptr* isolate			PCR results					Race[4]	MLHT
			Glenlea	6B662	6B365	*CHS1*	*ToxA*[1]	*ToxB*[2]	*toxb*[3]	*ToxC1*		
Tat-14-1	Tatarstan, Vysokogorsky district	Triticum aestivum, spring wheat variety KVS Torridon	R	R	R	+	+	-	-	+	4	*
Tat-14-2			N	R	R	+	+	-	-	+	2	I
Tat-14-3			R	R	C	+	+	-	-	+	3	*
Tat-14-4			N	R	C	+	+	-	-	+	1	*
Tat-14-5			N	R	C	+	-	-	-	+	1	I
Tat-15-1	Tatarstan, Tyulyachinsky district	T. aestivum, winter wheat, v. Vasilievna	N	R	C	+	-	-	-	+	1	J
Tat-15-2			R	R	R	+	-	-	-	+	4	J
Tat-16-1	Tatarstan, Verkhneuslonsky District	T. aestivum, winter wheat, variety unknown	N	C	R	+	+	-	-	+	7	*
Tat-16-2			N	R	R	+	-	-	-	+	2	L
Tat-16-3			N	R	C	+	+	-	-	+	1	*
Tat-16-4			N	R	C	+	+	-	-	+	1	*
Tat-16-5			N	R	C	+	+	-	-	+	1	*
Tat-16-6			N	C	C	+	+	-	-	+	8	*
Tat-16-7			N	C	C	+	-	-	-	+	8	*
Tat-16-8			N	R	C	+	+	-	-	+	1	*
Tat-16-9			N	R	C	+	+	-	-	+	1	*
Tat-16-10			N	C	C	+	+	-	-	+	8	L
Tat-19-1	Tatarstan, Chistopol district	T. aestivum, winter wheat, variety unknown	N	R	R	+	+	-	-	+	2	*
Tat-19-2			R	R	R	+	+	-	-	+	4	*
Tat-19-3			N	R	C	+	+	-	-	+	1	G
Tat-19-4			N	R	C	+	+	-	-	+	1	G
Tat-19-5			N	R	C	+	+	-	-	+	1	*
Tat-19-6			N	R	C	+	+	-	-	+	1	*
Tat-19-7			N	R	C	+	+	-	-	+	1	*
Tat-19-8			N	R	C	+	+	-	-	+	1	*
Pen-11-1		T. aestivum,	N	R	R	+	+	-	-	+	2	E

Pen-11-2	Penza, field of agricultural institute	winter wheat, v. Ekada 279	N	R	R	+	+	-	-	+	2	E
Pen-11-3			N	R	R	+	+	-	-	+	2	E
Pen-11-4			N	R	R	+	+	-	-	+	2	E
Pen-11-5			N	R	R	+	+	-	-	+	2	*
Pen-13-1		breeding line	R	R	C	+	+	-	-	+	3	*
Pen-14-1		T. aestivum, winter wheat, breeding line	R	R	C	+	+	-	-	+	3	*
Pen-14-2			N	C	C	+	+	-	-	+	8	*
Pen-14-3			N	R	C	+	+	-	-	+	1	*
Pen-14-4			N	R	C	+	+	-	-	+	1	*
Pen-14-5			R	R	C	+	+	-	-	+	3	*
Pen-15-1		T. aestivum, winter wheat, v. Nastavnik	N	C	C	+	+	-	-	+	8	*
Pen-15-2			R	R	C	+	+	-	-	+	3	*
Pen-15-3			R	R	R	+	+	-	-	+	4	C
Pen-15-4			N	C	C	+	+	-	-	+	8	C
Pen-15-5			R	R	C	+	+	-	-	+	3	C
Pen-15-6			R	R	C	+	+	-	-	+	3	C
Pen-16-1		T. aestivum, winter wheat, v. Lutescens	N	R	R	+	+	-	-	+	2	*
Pen-16-2			R	R	R	+	+	-	-	+	4	D
Pen-16-3			R	R	R	+	+	-	-	+	4	D
Pen-16-4			R	R	R	+	+	-	-	+	4	*
Pen-16-5			R	C	C	+	+	-	-	+	6	*
Pen-21-1		T. aestivum, winter wheat, breeding line	R	R	R	+	-	-	-	+	4	*
Pen-22-1			R	R	C	+	+	-	-	+	3	K
Pen-22-2			R	R	C	+	+	-	-	+	3	K
Pen-22-3			R	R	R	+	+	-	-	+	4	*
Sar-12-1	Saratov Region, Arkadak District	Triticum durum, spring wheat, v. Saratovskaya Zolotistaya	R	R	R	+	-	-	-	+	4	*
Sar-12-2			N	R	C	+	+	-	-	+	1	*
Sar-12-6			N	R	R	+	+	-	+	+	2	*
Sar-12-20			N	R	C	+	-	-	-	+	1	*
Sar-12-21			N	R	C	+	+	-	-	+	1	*
Sar-12-22			N	R	C	+	+	-	-	+	1	*
Sar-12-23			R	R	C	+	+	-	-	+	3	*

Sar-12-24			R	R	R	+	+	-	-	+	4	F
Sar-12-25			R	R	R	+	+	-	-	+	4	F
Sar-14-1	Saratov Region, Arkadak District	T. durum, spring wheat, v. Nik	N	R	C	+	-	-	+	+	1	*
Sar-14-4			R	R	R	+	-	-	-	+	4	*
Sar-14-5			N	R	C	+	-	-	-	+	1	*
Sar-14-6			N	C	R	+	-	-	-	+	7	*
Sar-14-7			N	R	C	+	-	-	-	+	1	*
Sar-14-8			N	R	C	+	+	-	-	+	1	H
Sar-14-9			R	R	R	+	+	-	-	+	4	H
Sar-15-1	Saratov Region, Arkadak District,	T. durum, spring wheat, v. In Memory of Vasilchuk	N	R	R	+	+	-	-	+	2	B
Sar-15-2			N	R	C	+	+	-	-	+	1	B
Sar-15-3			N	R	C	+	+	-	+	+	1	A
Sar-15-4			N	R	C	+	+	-	-	+	1	A
Sar-15-6			R	R	R	+	+	-	-	+	4	B
Sar-15-7			N	C	R	+	+	-	-	+	7	B
Sar-15-8			N	R	R	+	+	-	-	+	2	B

*unique multilocus haplotypes (MLHT) by SSR markers; A-K – Ptr clones. R – resistance reaction; N – necrosis; C – chlorosis
1 – the same amplification result was obtained with primers Ta51F/Ta52R [34] and primers specific for the ORF of the ToxA gene ToxA1/ToxA2 [14]; 2 – the same amplification result was obtained with primers TB71/TB06 [34] and primers specific for the ORF of the ToxB gene ToxB1/ToxB2 [14]; 3 – the same amplification result with primers TB71/TB58 [34], 90-2F1/90-2R1 [14] and toxbF/toxbR [35]; 4 - according to phytopathological analysis.
