# Supplementary material for: Genetic Diversity of Pyrenophora tritici-repentis Based on Virulence Phenotyping, Effector Gene Profiling, and SSR Markers
Source: J Fungi (Basel). 2026 Oct 20;12(10):787. doi: 10.3390/jof12100787 (PMC13705791; doi:10.3390/jof12100787)
Supplement: Supplementary file 1 [file jof-12-00787-s001.zip › Table S2.pdf]

Table S2. Sequences of primers used in the work.

Primers	Sequences (5'–3')	Gene	Ta*	PCR product size (bp)	Reference
CHS-79F	TGGGGCAAGGATGCTTGGAAGAAG	CHS-1	58°	275	[34]
CHS-354R	TGGAAGAACCATCTGTGAGAGTTG				
TA51F	GCGTTCTATCCTCGTACTTC	ToxA		573	
TA52R	GCATTCTCCAATTTTCACG				
TB71	GCTACTTGCTGTGGCTATC	toxb		232	
TB58	TATGAATGATTGACTGGGGTTA				
TB71	GCTACTTGCTGTGGCTATC	ToxB		232	
TB06	ACGTCCTCCACTTTGCACACTCTC				
ToxA1	GTCATGCGTTCTATCCTCG	*ToxA* (*ORF*)	55°	294	[14]
ToxA2	CCTATAGCACCAGGTCGTCC				
ToxB1	GACTACCATGCTACTTGCTGTG	*ToxB* (*ORF*)		245	
ToxB2	AACAACGTCCTCCACTTTGC				
90-2F1	AAGTGGTCATTGCGACTGG	toxb		157	
90-2R1	CCTCCACTTGCCAAACTCTC				
PtrUniqueF2	GGACTTTGGCTTTCTATTGTGC	–**	58°	490	[31]
PtrUniqueR2	CTTGGTGAATGGTGAAGATGG				
toxb F	TGTTTAATCTTATACTAATCCCTTG	toxb	55°	267	[35]
toxb R	TCTTGTAGCGCCTAAACTCT				
M4_13157 F	GAGCAGCATTTTGACGAGTG	ToxC1	60°	505	[6]
M4_13157 R	TGGAAGTCGTTCATTGTTGC				

Note: *annealing temperature, **species-specific DNA fragment.
