# Supplementary material for: Genetic Diversity of Pyrenophora tritici-repentis Based on Virulence Phenotyping, Effector Gene Profiling, and SSR Markers
Source: J Fungi (Basel). 2026 Oct 20;12(10):787. doi: 10.3390/jof12100787 (PMC13705791; doi:10.3390/jof12100787)
Supplement: Supplementary file 1 [file jof-12-00787-s001.zip › Table S3.pdf]

Table S3. Primers for microsatellites of *Pyrenophora tritici-repentis* used in the work [24].

Locus, repeat motif	Chromosome	Primers (5'→3')
PtrSSR01<br>$(AAC)_{32}$	1	F: TTTGGGAGATGGGGGAAA<br>R: TTGCGTCTGTGCGACATG
PtrSSR03<br>$(AG)_{25}$	1	F: ACGTCCCTGCTTACGGATGT<br>R: ATACCAAGCTTCTCCGCCTCT
PtrSSR05<br>$(AAG)_{26}$	2	F: GCGTGATCTCGCCACATATTA<br>R: CGACGTCCTTCACAGGATTTA
PtrSSR07<br>$(AGT)_{11}$ACGT TGT$(AGT)_{12}$	8	F: ATCCACCTCCGTTGCAGTT<br>R: CTGATTTCACCGCGAAAACA
PtrSSR09<br>$(AGT)_{31}$	4	F: CCACGCTCATCACTTTGTCTA<br>R: CCACTTGATTGAGTTTTGCG
PtrSSR12<br>$(AAG)_{18}$	6	F: AAGAGGTGTCGACTAGCGTTT<br>R: GGCTTAATTTTAAGCGCGTG
PtrSSR13<br>$(AAG)_{23}$	7	F: TCGTGGGTATAAAACGGCTCT<br>R: TTCGGCGGCTTCATATTACT
PtrSSR14<br>$(AAT)_{20}$	7	F: TAGAGCCTGCCGAGATTGTTT<br>R: GCCTTCGGCCAAAGTCATA
PtrSSR15<br>$(AAG)_{37}$	8	F: CGCCAACATAGTTGCGAATT<br>R: TCCGCCTCTGTAGGTGTTAAA
PtrSSR16<br>$(AGT)_{37}$	8	F: TTACGATGCGCTCACACTAGA<br>R: ATGGCCCGCTTGTAGTCTTTA
