# Supplementary material for: Genetic Diversity of Pyrenophora tritici-repentis Based on Virulence Phenotyping, Effector Gene Profiling, and SSR Markers
Source: J Fungi (Basel). 2026 Oct 20;12(10):787. doi: 10.3390/jof12100787 (PMC13705791; doi:10.3390/jof12100787)
Supplement: Supplementary file 1 [file jof-12-00787-s001.zip › Table S4.pdf]

Table S4. Observed allele frequencies of ten microsatellite loci in samples of the Volga population of *Pyrenophora tritici-repentis*.

Locus	Allele, bp	Allele frequency in a population sample (number of isolates)		
		Tat (25)	Pen (25)	Sar (24)
SSR13	300	0,840	0,192	0,333
	320	0,120	0,000	0,125
	350	0,040	0,269	0,167
	400	0,000	0,423	0,292
	420	0,000	0,077	0,000
	450	0,000	0,038	0,083
SSR07	170	0,000	0,115	0,000
	190	0,120	0,000	0,000
	200	0,200	0,000	0,000
	210	0,040	0,000	0,083
	230	0,000	0,269	0,208
	240	0,120	0,038	0,125
	250	0,400	0,462	0,000
	280	0,040	0,115	0,292
	300	0,000	0,000	0,042
	350	0,000	0,000	0,167
	400	0,080	0,000	0,083
SSR14	200	0,400	0,115	0,750
	220	0,520	0,615	0,250
	250	0,080	0,269	0,000
SSR05	200	0,000	0,154	0,000
	230	0,040	0,154	0,000
	240	0,120	0,231	0,458
	250	0,160	0,346	0,250
	260	0,440	0,038	0,208
	280	0,240	0,077	0,083
SSR01	230	0,440	0,609	0,583
	250	0,560	0,391	0,417
SSR15	150	0,000	0,038	0,000
	200	0,200	0,000	0,000
	220	0,440	0,077	0,000
	250	0,000	0,346	0,625
	270	0,000	0,308	0,125
	300	0,360	0,231	0,000
	350	0,000	0,000	0,083
	400	0,000	0,000	0,167
SSR03	250	0,320	0,435	1,000
	260	0,440	0,478	0,000
	270	0,160	0,043	0,000
	300	0,080	0,043	0,000
SSR16	200	0,040	0,000	0,000
	250	0,240	0,308	0,000
	300	0,440	0,269	0,042
	350	0,000	0,192	0,000
	400	0,160	0,000	0,292

	450	0,120	0,077	0,667
	500	0,000	0,077	0,000
	550	0,000	0,077	0,000
SSR12	250	0,440	0,769	0,125
	270	0,240	0,077	0,000
	300	0,320	0,154	0,875
SSR09	250	0,680	0,500	0,565
	270	0,200	0,462	0,304
	300	0,120	0,038	0,130
